# Sarcoidosis Incidentally Diagnosed: A Case Report

**DOI:** 10.1155/2014/702868

**Published:** 2014-07-08

**Authors:** Besir Kesici, Ahmet Burak Toros, Levent Bayraktar, Adem Dervisoglu

**Affiliations:** ^1^Department of Gastroenterohepatology, Medical Faculty Bahcesehir University, Istanbul, Turkey; ^2^Department of Radiology, Medical Park Fatih Hospital, Istanbul, Turkey; ^3^Department of General Surgery, Medical Faculty Bahcesehir University, Istanbul, Turkey

## Abstract

Sarcoidosis is a chronic, granulomatous condition with unknown cause. Because most of the patients are free of clinical symptoms, sarcoidosis should be considered in differential diagnosis if noncaseous granuloma is noted in biopsies, performed for other reasons. With no clinical symptoms, our patient was diagnosed with sarcoidosis upon identifying noncaseous granuloma in the lymph node biopsy material collected during the laparoscopic operation, performed for gallbladder polyp.

## 1. Introduction

Sarcoidosis can be seen in all races, at all ages, and in both sexes. Its prevalence varies by the organs involved, severity of the disease, and clinical course, as well as among populations and races [1, 2]. The prevalence is higher in Afro-Americans and in the Scandinavian race. The incidence of sarcoidosis in Turkey is estimated to be 4/100.000 [3]. Etiological factors leading to sarcoidosis are not clear yet [1]. The typical histopathologic lesion of sarcoidosis is granuloma without caseous necrosis [2]. Almost half of the patients with sarcoidosis present with no symptoms. Diagnosis in some cases is made with the help of findings observed in chest X-ray taken for other reasons. Since the lungs are the most frequently involved organ (90–95%), patients usually present with pulmonary complaints (e.g., shortness of breath, dry cough) [1, 2, 4]. Some cases, on the other hand, might be seeking medical attention for constitutional symptoms or signs of extrapulmonary site involvement [1]. One-third of the cases may present with nonspecific complaints such as fatigue, weakness, easy tiredness, and weight loss. Nocturnal sweating may rarely be present. Fever may persist for weeks and be 40°C in rare cases [4]. Diagnosis is based on pathologic demonstration of granulomas without caseous necrosis in the presence of clinical and radiological findings consistent with sarcoidosis, where other causes which might be associated with a similar manifestation are ruled out [1, 4].

## 2. Case Report

The 20-year-old female patient presented with abdominal pain, which began 10 days ago, and underwent relevant examinations, among which abdominal USG demonstrated a polypoid lesion with a diameter of 5.7 mm in the gallbladder, and laparoscopic cholecystectomy was therefore performed. Plaques, white in color, on the liver were noted during the surgery, although biopsy material was not collected, due to the risk of bleeding. The patient was referred to our clinic when chronic cholecystitis and nonnecrotizing granulomatous lymphadenitis in one lymph node were identified in the sections. Patient's investigations yielded normal hepatic enzymes; HBsAg and AntiHCV were negative. PA chest X-ray demonstrated fullness at both hili. Thoracic CT was performed (Figures 2, 3, 4, and 5) and was reported as upper mediastinal-bilateral superior and inferior paratracheal-aortopulmonary-paraesophageal-subcranial LAMs, the largest of which has a diameter of up to 13 mm, are observed. Pleuroparenchymal sequelae densities are present in the apical lobe of bilateral lungs. Centrilobular nodules are observed at bilateral lungs. There are celiac-periportal LAMs in the section crossing the upper abdomen. The findings reported principally suggest sarcoidosis. Patient's biopsies were therefore reevaluated at the pathology clinic of the university hospital. Nonnecrotizing granulomatous lymphadenitis was identified at the lymph node. The findings suggested sarcoidosis. Increased levels of angiotensin-converting enzyme (ACE) were found (197,5 U/L; normal range: 8–52). Calcium level was normal.

## 3. Discussion

Sarcoidosis is a chronic, granulomatous condition with unknown cause [5]. Diagnosis is based on tissue biopsies, demonstrating granulomas without caseous necrosis in the presence of clinical and radiological findings, consistent with sarcoidosis, where other causes which might be associated with a similar manifestation are ruled out [1, 4]. Presence of granulomas alone is not diagnostic. From the histological perspective, same lesions may also be observed in chronic berylliosis, tuberculosis, histoplasmosis, coccidioidomycosis, lymphoma, Hodgkin's disease, bronchogenic carcinoma, foreign body granuloma, schistosomiasis, syphilis, and leprosy [6].

Clinical signs depend on disease duration, organ involvement, extent of involvement, and the activity of the granulomatous event.

Lungs are the most frequently involved organ. Half of the patients are asymptomatic and are usually diagnosed when investigating for another cause. Bilateral hilar lymphadenopathy is characteristic and usually accompanied by paratracheal lymphadenopathy [7]. Our patient was diagnosed upon identifying noncaseous granuloma in the lymph node biopsy material, collected during the operation performed for gallbladder polyp.

Serum ACE is increased in 30 to 80% of patients with sarcoidosis [8]. This variation is considered to be associated with measurements performed at different timeframes and different radiological stages as well as with the presence of extrapulmonary lesions [9, 10]. There are contradicting data on the disease and activity relationship. In our patient, the disease was clinically inactive despite the increased ACE level.

**Figure 1:**
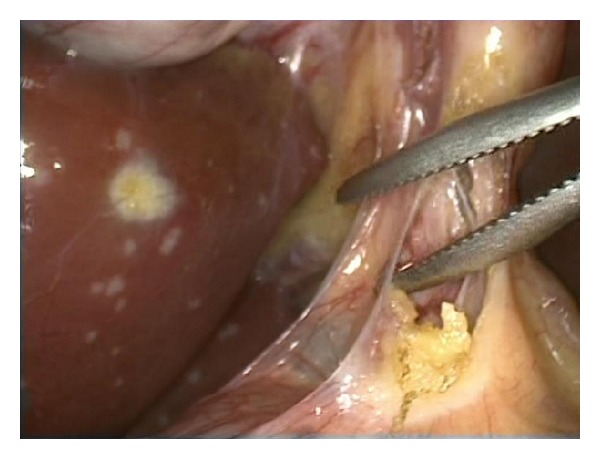
White plaques on the liver, noted during laparoscopy.

**Figure 2:**
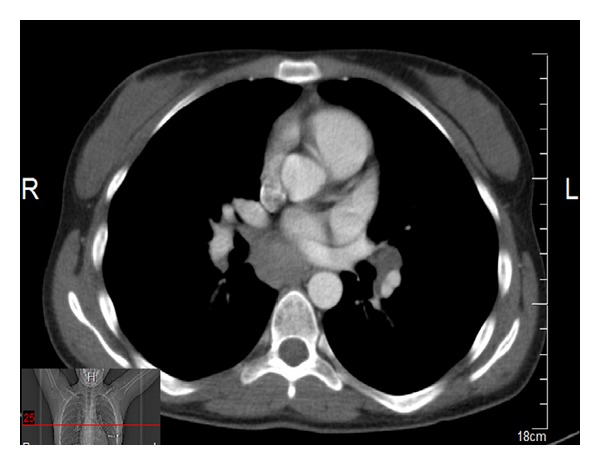
Thoracic CT views.

**Figure 3:**
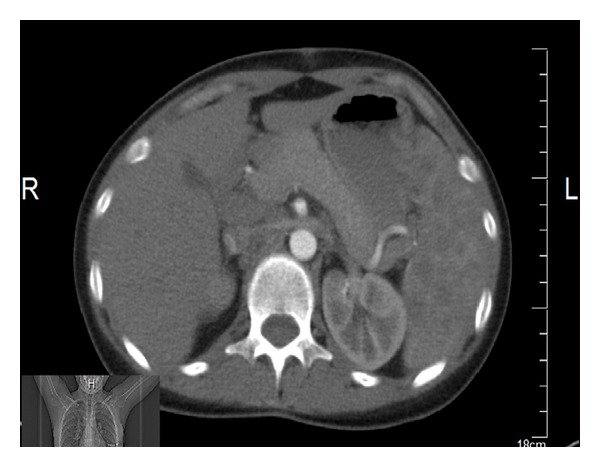
Thoracic CT views.

**Figure 4:**
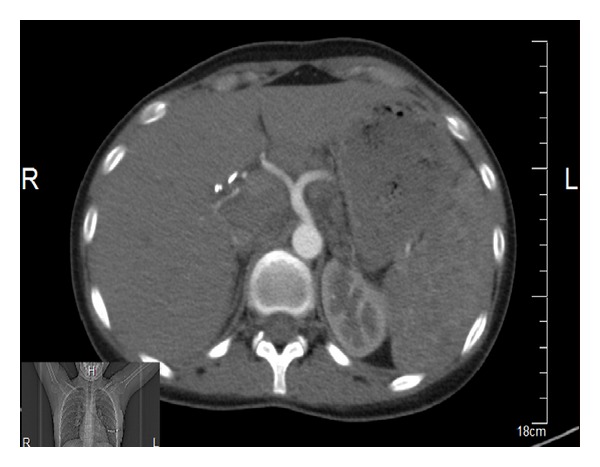
Thoracic CT views.

**Figure 5:**
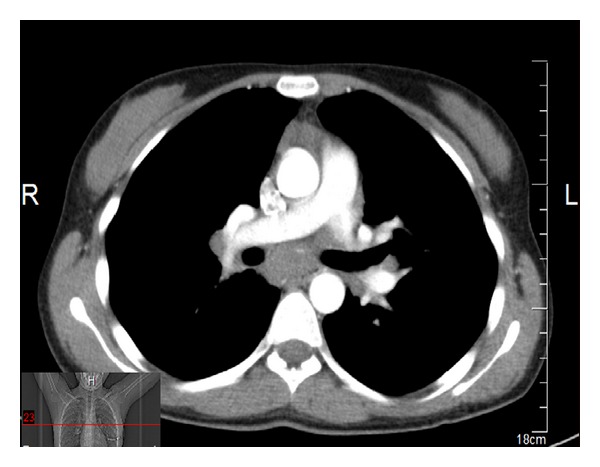
Thoracic CT views.

Although hepatic involvement is seen in 75% of the cases in sarcoidosis, clinical symptoms and impairment of the hepatic function are not common [11]. Plaques forming in the liver usually go unnoticed with imaging techniques. The nodules, which in rare cases reach to a diameter of 2 cm, may be mistaken for metastasis [12]. Similarly, white plaques were noted on the liver surface during our patient's surgery (Figure 1). On the other hand, liver was in normal appearance with abdominal USG and CT.

Sarcoidosis has a variable clinical course and prognosis. One-third of the patients undergo spontaneous remission. In 10–30% of the patients, however, the disease takes a chronic course [13, 14]. Corticosteroids remain as the main therapeutic choice [15]. Because our patient had no clinically relevant complaints, it was decided to follow up the patient without treatment (in accordance with a pulmonologist, who also performed on the patient a breath function test).
